# Protein-Protein Interactions in *Candida albicans*

**DOI:** 10.3389/fmicb.2019.01792

**Published:** 2019-08-07

**Authors:** Floris Schoeters, Patrick Van Dijck

**Affiliations:** ^1^VIB-KU Leuven Center for Microbiology, Leuven, Belgium; ^2^Laboratory of Molecular Cell Biology, Institute of Botany and Microbiology, KU Leuven, Leuven, Belgium

**Keywords:** *Candida albicans*, protein-protein interactions, *Candida* two-hybrid system, yeast two-hybrid system, BioGRID, *S. cerevisiae*

## Abstract

Despite being one of the most important human fungal pathogens, *Candida albicans* has not been studied extensively at the level of protein-protein interactions (PPIs) and data on PPIs are not readily available in online databases. In January 2018, the database called “Biological General Repository for Interaction Datasets (BioGRID)” that contains the most PPIs for *C. albicans*, only documented 188 physical or direct PPIs (release 3.4.156) while several more can be found in the literature. Other databases such as the String database, the Molecular INTeraction Database (MINT), and the Database for Interacting Proteins (DIP) database contain even fewer interactions or do not even include *C. albicans* as a searchable term. Because of the non-canonical codon usage of *C. albicans* where CUG is translated as serine rather than leucine, it is often problematic to use the yeast two-hybrid system in *Saccharomyces cerevisiae* to study *C. albicans* PPIs. However, studying PPIs is crucial to gain a thorough understanding of the function of proteins, biological processes and pathways. PPIs can also be potential drug targets. To aid in creating PPI networks and updating the BioGRID, we performed an exhaustive literature search in order to provide, in an accessible format, a more extensive list of known PPIs in *C. albicans*.

## Introduction

### Fungal Infections and *Candida albicans*

One to five million fungal species are estimated to exist of which only 400–600 (< 0.1%) are documented to be pathogenic to humans and of those only about 100 are commonly found as human pathogens (Taylor et al., 2001; Blackwell, 2011; de Pauw, 2011; Köhler et al., 2014; Kastora et al., 2017). However, these fungal pathogens/infections are still often overlooked and underestimated even though they have evolved from uncommon to a major global health problem, paradoxically due to the introduction of new medical therapies (Brown et al., 2012; Köhler et al., 2014; Editorial, 2017). Recent outbreaks have drawn more attention to fungal infections (Benedict et al., 2017). It is estimated that over a billion people are affected by superficial fungal infections and about 1.5 million people die due to invasive fungal diseases worldwide each year (Brown et al., 2012; Bongomin et al., 2017). Only a few classes of antifungals are available for the treatment of invasive infections and resistance is on the rise (Fairlamb et al., 2016; Editorial, 2017; Perlin, 2017). These invasive fungal infections are associated with high mortality rates (up to 50%) and occur mainly in patients who had major surgery, are immunocompromised or on heavy antibiotic treatments. However, major infections in healthy individuals are increasing. The majority of those fungal-related deaths are attributed to only four genera of fungi: *Cryptococcus*, *Aspergillus*, *Pneumocystis*, and *Candida* (Spellberg, 2008; Brown et al., 2012; Editorial, 2017). Specifically *Candida* infections are the 4th most common cause of hospital-acquired infections (Brown et al., 2012; Noble et al., 2017) and in the United States alone candidiasis is estimated to cost up to $2–4 billion yearly (Uppuluri et al., 2017). Within the genus *Candida*, *Candida albicans* is currently the most medically important species (Brown et al., 2012) but *Candida auris* is gaining a lot of recent publicity (see below).

*Candida albicans* is a pleiomorphic diploid fungus generally only found in humans. Up to 70% of humans are hosts of this fungus (Noble et al., 2017). It is generally considered as a commensal, but it can turn pathogenic in certain circumstances. These circumstances are often regarded as “caused” or provided by the host (e.g., being immunocompromised or taking antibiotic treatments) and not so much as actively generated by *C. albicans* itself (Köhler et al., 2014; Mitchell, 2016; Noble et al., 2017). *C. albicans* can cause superficial mucosal infections such as oral thrush or vulvovaginal candidiasis but also life-threatening invasive infections (Spellberg, 2008; Brown et al., 2012; Köhler et al., 2014; Noble et al., 2017). This switch from a commensal to a potentially lethal pathogen is still not fully understood (Noble et al., 2017). Recently, the 1st fungal cytolytic peptide toxin in *C. albicans*, candidalysin, was described and is thought to play a pivotal role in the pathogenicity of *C. albicans* (Mitchell, 2016; Moyes et al., 2016). *C. albicans* can thrive in the human body. It is able to evade the immune system, colonize every organ and form biofilms on implanted medical devices (Brown et al., 2012; Mathé and Van Dijck, 2013; Noble et al., 2017; Sherrington et al., 2017). It is not surprising that it is used as a model organism to study fungal pathogenesis (Kabir et al., 2012). *C. albicans* has a few remarkable characteristics that make it challenging to study. It is a diploid organism without a complete sexual cycle (no meiosis found so far) and has a non-canonical codon usage (CUG translated as serine rather than leucine), in addition, it lacks stable episomal plasmids and has a low transformation efficiency (Datta et al., 1990; Murad et al., 2000; Noble and Johnson, 2007; Stynen et al., 2010; Hickman et al., 2015). In recent years, as novel research tools have been developed, the whole genome has been sequenced and the ORFs have been made available for the community in Gateway^TM^-adapted vectors (Jones et al., 2004; Kaplanek et al., 2006; Muzzey et al., 2013; Legrand et al., 2018).

One important feature to study in order to understand an organism is its protein-protein interactions (PPIs) (Auerbach et al., 2002; De Las Rivas and Fontanillo, 2010; Khan et al., 2011). Advances in high-throughput detection techniques mean that mapping large physical PPI networks has become a possibility for several organisms (Auerbach et al., 2002; Schächter, 2002; Uetz, 2002; De Las Rivas and Fontanillo, 2010), which will help in compiling the interactome of the studied organisms (Bonetta, 2010; De Las Rivas and Fontanillo, 2010). Several techniques are available to study PPIs (von Mering et al., 2002; Khan et al., 2011; Rao et al., 2014; Podobnik et al., 2016) but the most prominent techniques to study PPIs on a high-throughput scale are the tandem-affinity purification (TAP) followed by mass spectrometry (Gingras et al., 2005; Krogan et al., 2006; De Las Rivas and Fontanillo, 2010) and the yeast two-hybrid (Y2H) assay (Uetz, 2002; De Las Rivas and Fontanillo, 2010; Xing et al., 2016). There are, however, about 25,000 CUG codons in *C. albicans*, complicating the use of *Saccharomyces cerevisiae* as a host organism to study *C. albicans* PPIs using the Y2H technique (Skrzypek et al., 2017). A possible solution is to change the CUG codons (Hoppen et al., 2007; Badrane et al., 2008) or work with only parts of the protein (Xu and Mitchell, 2001) to minimize translation problems, yet most researchers did not change the CUG codons when performing experiments in *S. cerevisiae* (Oughtred et al., 2016, 2018; Chatr-Aryamontri et al., 2017). In one study, it was observed that changing the CUG codons led to the discovery of a PPI not found when using non-altered CUG codons (Feng et al., 2017). An adapted *Candida* two-hybrid (C2H) system was developed to overcome the problem with the non-canonical codon usage and the first small-scale high-throughput screen was performed with this system (Stynen et al., 2010; Schoeters et al., 2018). The TAP-tag has also been used to study PPIs in *C. albicans*, but only a limited number of PPIs or complexes have been studied with this technique (see below).

## Results

### Databases to Store PPI Data and Curation of the Literature Describing PPIs Demonstrated in *C. albicans*

Several large-scale chromatin immunoprecipitation (ChiP) studies have already been done on *C. albicans* to study protein-DNA interactions, leading to several networks (Zordan et al., 2007; Tuch et al., 2008; Nobile et al., 2012; Hernday et al., 2013; Znaidi et al., 2013) and even leading to a few PPIs in *C. albicans* (Znaidi et al., 2013). However, limited by the difficulties encountered in *C. albicans* research (Noble and Johnson, 2007; Palzer et al., 2013) only a small number of PPIs have been detected in *C. albicans*. In contrast to the ChiP studies, no large scale high-throughput PPI screens have been performed for *C. albicans* and only a limited number of papers have described PPIs in *C. albicans* (Wang et al., 2014; Chatr-Aryamontri et al., 2017; Márkus et al., 2017; Oughtred et al., 2018). Several (public) databases are available to screen for PPIs, but they are extremely limited when it comes to *C. albicans* PPIs. The Search Tool for the Retrieval of Interacting Genes/Proteins (STRING) database does not even mention *C. albicans* (Szklarczyk et al., 2017) and the Database for Interacting Proteins (DIP) only holds a handful interactions (Salwinski et al., 2004). Another database, The Molecular INTeraction Database (MINT), only mentions 24 PPIs for *C. albicans* (Licata et al., 2012). The IntAct MINT mentions 25 interaction protein pairs (Kerrien et al., 2012). The Biological General Repository for Interaction Datasets (BioGRID) contained 188 PPIs extracted from 42 publications, release 3.4.156 (January 2018). These 188 interactions (using 139 unique genes) can further be divided into 128 unique protein interacting pairs (non-redundant interactions) (Chatr-Aryamontri et al., 2017; Oughtred et al., 2018). Because the BioGRID contains the largest number of PPIs for *C. albicans*, we decided to further continue our work with the BioGRID. A literature search revealed many PPIs that were not yet mentioned in this database. The absence of many interactions from the literature in the BioGRID (and other databases) also shows that PPI data are not generally sent to databases such as the BioGRID. A remarkable example is that the BioGRID database, release 3.4.156, mentioned only one interaction that was found with crystallography (van den Berg et al., 2016) while several structures of *C. albicans* proteins or enzymes showing PPIs have already been studied with crystallography (Whitlow et al., 1997; Senay et al., 2003; Echt et al., 2004; Morgunova et al., 2007; Raczynska et al., 2007; Hast and Beese, 2008; Santini et al., 2008; Arachea et al., 2010; Rocha et al., 2011; Nakamura et al., 2013; Sheng et al., 2013; Yan et al., 2015; Nasser et al., 2016; Tonthat et al., 2016; Hong et al., 2017; Sinha and Rule, 2017; Dostal et al., 2018; Garcia et al., 2018; Kiliszek et al., 2019) and mentioned in the protein data bank^1^. A second observation during our literature search for PPIs in *C. albicans* is that certain “keywords” commonly used in papers studying PPIs are not often used by *C. albicans* researchers in their manuscripts.

To update the BioGRID with regard to *C. albicans* PPI data we performed an exhaustive literature search using several keywords (Table 1) in combination with “*C. albicans*” using Google search and PubMed. In addition to a literature search for novel PPIs we also checked the already available data for potential mistakes in order to correct them. All the novel interactions and/or mistakes in the already available data found during our literature search were sent to the BioGRID in order to update their data.

**TABLE 1:** Keywords used to search PubMed and Google.

Protein-protein interaction(s)
TAP tag
Co-IP
Western blot
Affinity purification
FRET
BRET
Yeast two hybrid
Y2H
Protein interaction(s)
Immunoprecipitation
Crystal structure
Physical interaction

*Every keyword was used in combination with “*C. albicans*”. E.g., “*C. albicans* Tap-tag” was used as a search term.*

### Putting PPIs in *C. albicans* in Perspective

If one compares the data for *C. albicans* with the data available for *S. cerevisiae* (up to 171,000 non-unique interactions) (von Mering et al., 2002; Oughtred et al., 2018) or certain bacteria (Parrish et al., 2007; Wang et al., 2010a; Oughtred et al., 2018), then it is easy to see that *C. albicans* PPI data are lagging behind significantly even though it is a highly studied organism. Another observation is that the golden standard reference website for the *Candida* community^2^ (Skrzypek et al., 2017, 2018) does not have a direct link or a file to PPIs in *C. albicans* in contrast to the yeast genome database^3^ that integrated, in a separate “tab”, all the interactions for a protein of interest mentioned on the BioGRID (Cherry et al., 2012). The combination “PPI” and “*C. albicans*” is also rarely used in the published literature (Table 2). This difference in found papers using the search term “PPI” and the name of the organism also shows that there is less work done regarding PPIs for *C. albicans* compared with the other four organisms (Table 2) and/or that *C. albicans* researchers are less inclined to use the term “PPIs” in their manuscripts. Four organisms were compared with *C. albicans*. *S. cerevisiae* and *Schizosaccharomyces pombe* were used since they are also yeast models. *Escherichia coli* is the bacterial “counterpart,” serving as a model organism for bacteria while *Arabidopsis thaliana* is a plant model.

**TABLE 2:** A search on pubmed (https://www.ncbi.nlm.nih.gov/pubmed/) using “PPIs” and the name of the organism resulted in X publications, while a search on the Biological General Repository for Interaction Datasets (https://wiki.thebiogrid.org/doku.php/statistics) shows how many PPIs are curated.

**Searched keywords on PubMed**	**Results PubMed search**	**Non-redundant PPIs in BioGRID November 2018 (Chatr-Aryamontri et al., 2017)**
Protein-protein interactions *C. albicans*	27 publications	611
Protein-protein interactions *S. pombe*	106 publications	9575
Protein-protein interactions *S. cerevisiae*	1714 publications	109 759
Protein-protein interactions *E. coli*	1740 publications	12 801
Protein-protein interactions *A. thaliana*	642 publications	35 897

The knowledge of PPIs is important for fully unraveling the complexity of organisms (De Las Rivas and Fontanillo, 2010). Besides the importance of PPIs for the fundamental understanding of an organism, they also form potential targets for specific drugs (Khan et al., 2011; Bakail and Ochsenbein, 2016; Nishikawa et al., 2016). The latter is very important since finding novel drug targets is hard due to the similarities between the eukaryotic pathogen and the eukaryotic host (Ismail et al., 2018). The currently limited availability of antifungals, rising resistance, and the increase of fungal infections underscore the need to identify new drug targets (Brown et al., 2012; Fairlamb et al., 2016; Perlin, 2017). PPIs might thus play an important role in the development of novel, very specific, antifungal drugs (Kingwell, 2016; Liu et al., 2018). An interesting example is blocking the interaction of Cdc42 with its CRIB-domain binding effectors and thereby inhibiting hyphal growth (Su et al., 2007).

Several techniques have been used to detect the interactions described for *C. albicans*. The majority of them were found using affinity-capture techniques or by using the traditional Y2H technique as described below. The most important techniques used to study PPIs in *C. albicans* are described below.

### Approaches to Study PPIs in *C. albicans*

Several techniques are available to detect PPIs (Rao et al., 2014; Podobnik et al., 2016), but only a select few have been adapted for use in *C. albicans* (Boysen et al., 2009; Stynen et al., 2010; Palzer et al., 2013; Subotić et al., 2017). For a complete overview of all the used techniques and detected protein interactions in *C. albicans*, we refer to the BioGRID website^4^ (Chatr-Aryamontri et al., 2017). So far, no large-scale or genome-wide screens have been performed, however, Prof. Whiteway’s lab has performed two tandem affinity purifications (TAP) and detected more than 200 PPIs (Tebbji et al., 2014, 2017). Prof. Liu’s lab has also used a TAP approach to identify 103 interacting proteins for the Wor1 protein (Alkafeef et al., 2018) while the labs of Profs. Emili, Gingras, and Cowen found more than 250 PPIs when studying Hsp90 (O’Meara et al., 2019). The labs of Profs. Dickman and Sudbery used the Stable Isotope Labeling with Amino Acids in Cell Culture (SILAC) technique combined with mass-spectrometry (MS) and identified 126 interacting proteins for Cdc14 (Kaneva et al., 2019).

**Co-immunoprecipitation (Co-IP)** is a type of affinity purification technique that is referred to as “affinity-western” in the BioGRID database (Chatr-Aryamontri et al., 2017; Oughtred et al., 2018). Many of the studied interactions with the classic Co-IP technique were experiments to validate interactions found with other techniques such as the Y2H technique (Ni et al., 2004; Fang and Wang, 2006; Kaneko et al., 2006; Berggård et al., 2007; Hoppen et al., 2007; Badrane et al., 2008; Lu et al., 2008; Lavoie et al., 2010; Sun et al., 2013; Lee H.J. et al., 2015). An expert with this technology is Prof. Wang who has used Co-IP a lot in his lab and showed several interactions (Zheng et al., 2004, 2007; Li et al., 2007, 2008, 2012; Sinha et al., 2007; Bai et al., 2011; Zeng et al., 2012; Wang et al., 2013, 2016; Gao et al., 2014; Huang et al., 2014a, b; Guan et al., 2015; Au Yong et al., 2016; Liu et al., 2016; Yao et al., 2016, 2017; Yang et al., 2018). Several tags have been used, such as a Flag-tag (Umeyama et al., 2002; Singh et al., 2011), Myc-tag (Cheetham et al., 2007, 2011; Sinha et al., 2007; Kos et al., 2016), GFP or derived tags (Bishop et al., 2010; Greig et al., 2015), HA-tag (Ni et al., 2004; Askew et al., 2011; Sellam et al., 2019), TAP-tag (see below) or the protein A tag with a TEV protease site (Blackwell et al., 2003). The Co-IP technique is rather limited and is not suited for high-throughput systems (Sun et al., 2013).

**Tandem-affinity purification** is another affinity purification technique that uses a TAP tag to perform a two-step specific affinity purification process. The original tag incorporated two protein A domains and the calmodulin binding peptide separated by a tobacco etch virus (TEV) protease site to provide the two-step affinity purification (Rigaut et al., 1999). Different versions of the TAP-tag were later designed without the TEV protease site (Xu et al., 2010). The TAP technique has the advantage that it can be used in a large-scale high-throughput setup where protein complexes are purified in two steps followed by protein identification with MS (Xu et al., 2010; Rao et al., 2014; Podobnik et al., 2016; O’Meara et al., 2019). It has, for example, been proven to be very efficient to perform large-scale screenings to identify PPIs and protein complexes in *S. cerevisiae* (Gavin et al., 2002; Krogan et al., 2006). A TAP tag was first successfully utilized in *C. albicans* by Kaneko et al. (2004) to purify the *C. albicans* septin protein complex (Kaneko et al., 2004), which was later confirmed by another group (Sinha et al., 2007). Both groups used a TAP-tagged Cdc11 protein and showed the same interactions except for two extra interactions found in the study from Sinha et al. (2007). One of these interactions was the Gin4 protein shown to interact with Cdc11. The Cdc11-Gin4 interaction was interestingly reported to be detected only after a 150 min hyphal induction of the cells, but not after a 10 min induction (Sinha et al., 2007). Kaneko et al. (2004) did not find this interaction, but they only induced hyphal growth for 90 min (Kaneko et al., 2004), showing the need to induce hyphal growth long enough to have Gin4 interact with the septin complex. Later studies not using a TAP tag approach also demonstrated the interaction of Gin4 with members of the septin complex (Li et al., 2012; Au Yong et al., 2016). The TAP-tag has also been applied to study several other complexes (Corvey et al., 2005; Lavoie et al., 2008; Blackwell and Brown, 2009; Ryan et al., 2012; Zhang et al., 2012; Tebbji et al., 2014, 2017; Guan et al., 2015; Lee J.E. et al., 2015; Rao et al., 2016; Alkafeef et al., 2018). The mediator complex was studied twice (Zhang et al., 2012; Tebbji et al., 2014) and both studies showed an overlap in found proteins (subunits) for the mediator complex, However, Tebbji et al. (2014) found a total of 179 proteins interacting with Med7 while Zhang et al. (2012) tagged Med8 and used a pre-purification with heparin sepharose to bind the intact mediator complex followed by a TAP and only purified the 25 subunits of the mediator complex itself (Zhang et al., 2012; Tebbji et al., 2014). Whether this difference is caused by the use of the pre-purification step is unclear. More recently O’Meara et al. (2019) applied the TAP-tag to identify well over 250 PPIs.

The TAP-tags can also be used in a type of Co-IP experiment, tagging both the bait and prey constructs and not using an MS approach to identify the preys (Kaneko et al., 2006; Singh et al., 2009; Chen and Noble, 2012; Shapiro et al., 2012; Li et al., 2017).

Using TAP-tag approaches with a two-step purification process has the advantage to produce cleaner protein samples for MS (Kaneko et al., 2004; Blackwell and Brown, 2009). However, single-step purification protocols followed by MS have also led to the discovery of several PPIs (Tseng et al., 2010; Li et al., 2012; Guan et al., 2015; Xie et al., 2017). Two of those studies used a GFP-tagged protein to purify the complexes (Li et al., 2012; Guan et al., 2015) while Xie et al. (2017) used a Flag-tagged Ydj1 protein (Xie et al., 2017). Tseng et al. (2010) used *E. coli*-expressed, His-tagged Cdc14 protein to pull down interacting proteins from a Cdc14 deletion mutant cell lysate (Tseng et al., 2010). Proteins found in a single-step purification approach should be confirmed with another technique (Tseng et al., 2010; Guan et al., 2015) as it often leads to false positives.

**SILAC** is a technique that takes advantage of the *in vivo* incorporation of non-radioactive isotope-labeled amino acids. It can be used to detect the up- or down-regulation of proteins. For this, growth medium is supplemented with a labeled amino acid that is then incorporated into the proteins of the cells grown on this medium (Ong et al., 2002). By subsequently mixing cells grown in this medium with cells grown in regular medium, lysing the cells, purifying the protein(s), and then digesting the purified proteins, the relative abundance of isotope-labeled and unlabeled proteins in the mixture can be determined by MS (Ong et al., 2002). In *C. albicans* the technique was first used in 2018 to perform a quantitative proteomic analysis of Cdc14 (Ong et al., 2002; Kaneva et al., 2018). Later the technique was used to study the proteome changes while transcriptionally repressing or pharmacologically inhibiting Hsp90 (O’Meara et al., 2019).

However, the technique can also be combined with an affinity purification step to identify PPIs. It was used to identify interacting proteins for Cdc14. This was achieved by growing a *C. albicans* strain with a phosphatase-dead, substrate-trapping Cdc14 protein with a Myc-tag (Cdc14^PD^-Myc, the “bait”) on medium supplemented with heavy isotope-labeled arginine and lysine (heavy medium). In parallel, a wild-type strain was grown on light medium. After mixing the bait and wild-type strain in a 1:1 ratio, the cells are lysed and the bait was pulled down followed by SDS-page and trypsin digestion. In the subsequent MS analysis, Cdc14-specific interacting proteins showed a heavy-to-light (H:L) ratio greater than 1:1 because specific interacting proteins will originate from the heavy medium, whereas non-specifically bound proteins will have a 1:1 ratio. A total of 126 interacting proteins for Cdc14 could be detected. Remarkably, only a few of the found interactions have also been demonstrated for the orthologous proteins in *S. cerevisiae* (Kaneva et al., 2019). See also Supplementary Tables S1, S2.

The SILAC approach (combined with affinity-purification) has several advantages: (1) it can be used in a high-throughput setup, (2) there is no forced cell localization (e.g., two-hybrid techniques force proteins into the nucleus), and (3) post-translational modifications can be preserved. The SILAC technique is also quantitative and not qualitative as is the traditional TAP-tag approach. The samples in SILAC are also analyzed as a whole single sample, thus minimizing the bias in sample handling (Ong et al., 2003; Emmott and Goodfellow, 2014).

**Bimolecular Fluorescence Complementation (BiFC)** is a technique that can be used to study PPIs *in vivo* in their native environment and location (Kerppola, 2008) and is one of many protein complementation techniques (PCA) (Zhou et al., 2011). Since its discovery, this technique has been applied in several organisms in a high-throughput setup (Miller et al., 2015). However, it proved difficult to apply this system in *C. albicans* and only recently it was used to detect several binary interactions (Mamouei et al., 2017; Subotić et al., 2017). Subotić et al. (2017) worked with an overexpression plasmid system with the genes under the control of the *MET3* promoter rather than endogenously tagged proteins. Mamouei et al. (2017) also used the *MET3* promoter, but were also able to use the native promoter for the Ftr1 and Fet34 BiFC constructs. Besides the need to codon-optimize the fluorophores (CUG codons), another potential explanation for the difficulties with adapting this system is the autofluorescence of *C. albicans* (Diaz et al., 2005; Graus et al., 2017). In addition to BiFC, several other PCAs are used for the detection of PPIs e.g., the split luciferase system (Stynen et al., 2012). However, so far, no other PCAs have been applied for PPI detection in *C. albicans* (Chatr-Aryamontri et al., 2017), despite the fact that several luciferases have been optimized for and used in *C. albicans*, for example, for the study of biofilm formation and drug susceptibility (Jacobsen et al., 2014; Kucharíková et al., 2015; Dorsaz et al., 2017). A split luciferase system can thus potentially be developed for use in *C. albicans*. It is also possible to use fluorophores and luciferases in a Förster Resonance Energy Transfer (FRET) or Bioluminescence Resonance Energy Transfer (BRET) system, however, BRET has so far not been reported in *C. albicans* (Chatr-Aryamontri et al., 2017). A FRET biosensor has been shown to work in *C. albicans* (Jain et al., 2018) and *Candida glabrata* (Demuyser et al., 2018).

**The Vesicle Capture Interaction (VCI)** assay was developed for use in *C. albicans* to circumvent the codon usage problem when using the model organism *S. cerevisiae*. VCI can be used to detect binary interactions. The technique is based on the targeting to endocytic vesicles by the Endosomal Sorting Complex Required for Transport (ESCRT) of which Snf7 (Vps32) is a subunit. The technique uses a *vps4*Δ mutant strain that promotes vesicular accumulation of Snf7. A bait protein is fused to the ESCRT subunit Snf7 while the prey protein is fused to a GFP protein. When bait and prey interact, a punctate GFP signal can be detected compared with a diffuse signal if no interaction occurs. The technique uses the native promoters so overexpression is avoided and real-time imaging facilitates the detection of transient interactions. The system was, so far as we know, only used in two studies from the lab that developed it (Boysen et al., 2009; Argimón et al., 2011). It remains an open question how applicable it is on a high-throughput setup.

**The expanded genetic code system** is a technique adapted for use in *C. albicans*. It relies on the incorporation of a synthetic photo-cross-linker amino acid, p-azido-L-phenylalanine (AzF), in a “bait” protein to covalently capture the binding partner (prey) after UV-activation. To incorporate AzF into the protein of interest, an amber stop codon needs to be introduced into this protein in a *C. albicans* strain that expresses the optimized orthogonal tRNA and tRNA synthetase for AzF. It is at the amber stop codon where AzF, provided in the medium, is incorporated (rather than terminating the translation). So far, only two interactions, TUP1 and TSA1 have been studied using this technique (Palzer et al., 2013). The technique has been proven to be valuable but has some limitations such as high dependence on the incorporation efficiency of AzF and the lack of site selectivity (Wang et al., 2009). The amounts of mutant protein is also reduced significantly compared with the wild-type (Palzer et al., 2013). The technique is still relatively new, and given its complexity and high cost, it seems unlikely that it will be used in a high-throughput setup soon. So far, no other studies applying this technique have been reported in *C. albicans*.

**The Yeast two-hybrid (Y2H)** system has become, since the first publication, one of the most used systems to detect PPIs *in vivo* (Auerbach et al., 2002; Silva et al., 2015). Several adaptations have been created and the Y2H system has been used to perform large-scale or even genome-scale PPI assays for several organisms (Legrain and Selig, 2000; Auerbach et al., 2002; Schächter, 2002; Hart et al., 2006). Compared to another technique often used in high-throughput screenings, mass spectrometry (MS) of purified complexes, the Y2H technique is easier and cheaper to use (von Mering et al., 2002; Brückner et al., 2009; Silva et al., 2015) and multiple commercial plasmids and yeast strains are available. It however, suffers from large amounts of false positives and negatives. The system also forces proteins into the nucleus, making it hard to use for certain proteins (e.g., cell membrane components) (Uetz et al., 2000; Silva et al., 2015). A potential solution for the problematic forced nuclear movement is the removal of parts of the protein of interest (Weber et al., 2002; Miwa et al., 2004). Despite these problems, it is still one of the most used and best techniques for high-throughput screening of PPIs (Silva et al., 2015).

In *C. albicans*, in spite of the codon usage problem, the Y2H technique is still responsible for the discovery of a large fraction of PPIs (Chatr-Aryamontri et al., 2017). Most researchers used the traditional Y2H system to detect PPIs (Gkourtsa et al., 2016; Chatr-Aryamontri et al., 2017; Oughtred et al., 2018) but it is also possible to use an adapted Y2H system: the SRYTH (Ste11p/Ste50p Related Yeast Two-Hybrid) system, which allows cytoplasmic PPI analysis (Mallick et al., 2016). This system makes use of the essential interaction of Ste11 and Ste50 to activate the high osmolarity glycerol (HOG) pathway in *S. cerevisiae*, in the absence of the SLN1–SSK1–SSK2/SSK22 pathway, in order to survive under osmotic stress (Wu et al., 2006; Mallick et al., 2016). Ste11 and Ste50 interact with each other through their sterile alpha motif (SAM) domain. These SAM domains can, however, be replaced by two proteins of interest (bait and prey). If those proteins interact, then Ste11 and Ste50 are brought together, the HOG pathway is activated and the cells survive under osmotic stress (Wu et al., 2006; Mallick et al., 2016). This system was used to study the mating pheromone pathway of *C. albicans* (Côte et al., 2011) and several transcription factors (Mallick and Whiteway, 2013).

**FIGURE 1:**
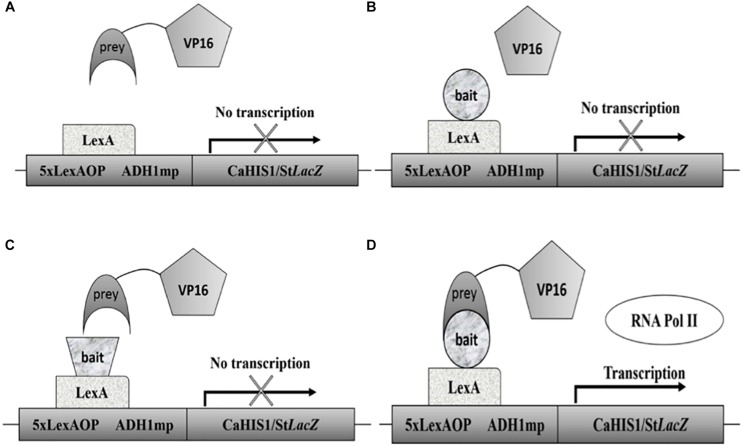
Schematic representation of the C2H system. **(A)** When no bait protein is attached to the DBD no interaction is possible and no transcription will take place. **(B)** Represents a situation in which no prey is attached to the AD leading to no transcription. In **(C)** both a prey and bait are present, but they do not interact so no transcription happens. Situation **(D)** depicts the interaction between a prey and bait protein thus leading to recruitment of RNA polymerase II and subsequent transcription. A common problem is the auto-activation by bait proteins. It is thus always essential to test auto-activation using the setup seen in part **B**.

**The Candida two-hybrid (C2H)** system is a special adaptation of the Y2H system (see Figure 1). It was designed in 2010 to address the codon usage problem in *S. cerevisiae*. Compared with the traditional Y2H system, it uses an integrative approach because plasmids are not very stable in *C. albicans*. Expression of the bait and prey constructs is driven by the *MET3* promoter and can be up-regulated by omitting methionine or both methionine and cysteine from the medium for a higher expression of the bait and prey constructs. With this system, several interactions were detected in a low-throughput setup (Stynen et al., 2010). The system was later used to confirm the interaction between the MAP kinases Cek1 and Cek2 (Correia et al., 2016), an interaction that is remarkably not found with the SRYTH system (Côte et al., 2011). The C2H system was also used to validate the PPIs found with the BiFC assay (Subotić et al., 2017). In the most recent paper using the C2H system, Wangsanut et al. (2018) tried to demonstrate the interaction between transcription factors Grf10 and Bas1 but without success (Wangsanut et al., 2018, 2019). The C2H system had been limited to small-scale studies until recently when it was adapted to a high-throughput setup (Legrand et al., 2018; Schoeters et al., 2018).

The CTG clade of *Candida* includes nine potential pathogens (Gabaldón et al., 2016). All of these can benefit from using the C2H system to study PPIs. One emerging pathogen in particular, *C. auris*, raises concerns due to its resistance against antifungals and sudden emergence (Lu et al., 2018). Table 3 lists the CTG clade species, all of which can be studied with the C2H system.

**TABLE 3:** Members of the CTG clade.

**Species**	
*Candida tenuis*	*Candida parapsilosis*
*Candida auris*	*Candida metapsilosis*
*Candida lusitaniae*	*Candida orthopsilosis*
*Metschnikowia fruticola 277*	*Candida sojae*
*Candida fermentati*	*Candida tropicalis*
*Candida guilliermondii*	*Candida dubliniensis*
*Debaryomyces fabryi*	*Candida albicans*
*Candida famata*	*Spathaspora passalidarum*
*Scheffersomyces stipitis*	*Lodderomyces elongisporus*
*Spathaspora arborariae*	

*All of these species translate the CUG codon to serine rather than leucine (Priest and Lorenz, 2015; Gabaldón et al., 2016) and can thus use the C2H system to study PPIs.*

### Comparing Data Between *C. albicans* and *S. cerevisiae*

*Saccharomyces cerevisiae* has been studied extensively regarding PPIs. Release 3.5.174, July 2019 from the BioGRID contains over 114,693 PPIs for *S. cerevisiae* while *C. albicans* only has 876 non-redundant interactions mentioned (Chatr-Aryamontri et al., 2017). While *S. cerevisiae* often functions as a model organism for fungi (in general) it is not always a good idea to extrapolate data from *S. cerevisiae* to *C. albicans*. While many pathways perform similar cellular functions, differences are also present, making it harder to simply extrapolate data from *S. cerevisiae* to construct pathways in *C. albicans* (Kobayashi and Cutler, 1998; Román et al., 2005, 2009; Bahn et al., 2007; Biswas et al., 2007; Cheetham et al., 2007; de Dios et al., 2010; Smith et al., 2010). Interestingly, of the 1,208 non-redundant PPIs in *C. albicans* (see Supplementary Tables S1, S2, based on release 3.5.174 from BioGRID and our literature search), we were able to find only 249 PPIs that were also demonstrated in *S. cerevisiae*. Several of the PPIs identified using Mcu1 and Snf6 as bait in *C. albicans* were not found in *S. cerevisiae* as it does not have orthologs of the two proteins according to the CGD database (based on BLAST analysis: query coverage of 13% between *Ca*Snf6 and *Sc*Snf6). Snf6 was however, identified as a member of the SWI/SNF complex and it was shown that the N-terminal domain, which interacts with Snf2, is conserved in *S. cerevisiae* (Tebbji et al., 2017); in addition, although Wor1, Hsp90, Med7, and Cdc14 together have over 500 interacting proteins in *C. albicans*, only a few of these interactions can be found with *S. cerevisiae* orthologs (Supplementary Tables S1, S2). The low overlap of PPIs between orthologs in *C. albicans* and *S. cerevisiae* might indicate big differences in PPIs and protein functions between the two organisms. This is no surprise given that *C. albicans* co-evolved with its host, being a commensal and potential pathogen, whereas *S. cerevisiae* is a saprophyte and only occasionally becomes pathogenic to humans. The two fungi are evolutionarily separated for 140–850 millions years (Kobayashi and Cutler, 1998; Biswas et al., 2007; Skrzypek et al., 2017) and only approximately 55% of the genes in both organisms have orthologs (we only looked at orthologous genes and not “best hits”) (Skrzypek et al., 2017). An important remark is that while in *S. cerevisiae* many interactions are studied with multiple techniques, this is not the case for *C. albicans*. Using different techniques is crucial to confirm PPIs (von Mering et al., 2002; Yu et al., 2008) so perhaps the low overlap is also partly due to the limited number of techniques used for detection of PPIs in *C. albicans* (see Supplementary Tables S1, S2).

An interesting, but often overlooked, dataset comprises the data of PPIs that were tested but could not be demonstrated as they are often not mentioned in the published literature. To further look into the differences between PPIs in *C. albicans* and *S. cerevisiae*, we also looked into the literature for PPIs that were investigated but not demonstrated for *C. albicans*. We then also compared this with known data in *S. cerevisiae*. With this information, we were able to construct Table 4. Our lab currently also hosts a more extensive and up-to-date list of interactions tested but not detected in *C. albicans* at: https://docs.google.com/spreadsheets/d/1nZDAPyyfCaqqtvAkU_Xt8wZcOPK6-17jJxQxKSen58g/edit#gid=93661881.

**TABLE 4:** A (limited) set of PPIs mentioned in several papers that could not be detected for *C. albicans* proteins.

***C. albicans (Oughtred et al., 2018)***			***S. cerevisiae (with orthologs) (Cherry et al., 2012)***	
**Bait**	**Prey**	**Technique**	**Y2H**	**Other technique(s)**
Far1	Ste11	Cyt. Y2H (Côte et al., 2011)	No	No
Far1	Ste11	Co-IP (Yi et al., 2011)	“	“
Far1	Hst7	Cyt. Y2H (Côte et al., 2011)	No	No
Far1	Hst7	Co-IP (Yi et al., 2011)	“	“
Far1	Cek1	Cyt. Y2H (Côte et al., 2011)	No	Yes
Far1	Cek1	Co-IP (Yi et al., 2011)	“	“
Far1	Cek2	Cyt. Y2H (Côte et al., 2011)	No	Yes
Far1	Cek2	Co-IP (Yi et al., 2011)	“	“
Cst5^∗^	Cek2^*^	Cyt. Y2H (Côte et al., 2011)	Yes	Yes
Ste11	Far1	Cyt. Y2H (Côte et al., 2011)	No	No
“	Ste11	Cyt. Y2H (Côte et al., 2011)	Yes	Yes
“	Cek2	Cyt. Y2H (Côte et al., 2011)	Yes	Yes
Hst7	Far1	Cyt. Y2H (Côte et al., 2011)	No	No
Hst7	Hst7	Cyt. Y2H (Côte et al., 2011)	Yes	Yes
Hst7^∗∗^	Cek1^∗∗^	Cyt. Y2H (Côte et al., 2011)	Yes	Yes
Hst7	Cek2	Cyt. Y2H (Côte et al., 2011)	“	“
Hst7	Cek2	C2H (Stynen et al., 2010)	“	“
Cek1	Far1	Cyt. Y2H (Côte et al., 2011)	No	Yes
Cek1°	Hst7°	Cyt. Y2H (Côte et al., 2011)	No	Yes
“	Cek1	Cyt. Y2H (Côte et al., 2011)	No	Yes
“	Cek2	Cyt. Y2H (Côte et al., 2011)	No	Yes
“	“	C2H (Stynen et al., 2010)	“	“
Cek2	Far1	Cyt. Y2H (Côte et al., 2011)	No	Yes
Cek2	Cst5	Cyt. Y2H (Côte et al., 2011)	No	Yes
“	Ste11	Cyt. Y2H (Côte et al., 2011)	Yes	Yes
Cek2	Hst7	Cyt. Y2H (Côte et al., 2011)	Yes	Yes
Cek2	Hst7	C2H (Stynen et al., 2010)	“	“
Cek2	Hst7	Y2H (Chen and Chen, 2001)		
Cek2°	Cek1°	Cyt. Y2H (Côte et al., 2011)	No	No
“	Cek2	Cyt. Y2H (Côte et al., 2011)	No	Yes
Gin4	Cdc24	Co-IP (Li et al., 2012)	No	No
“	Cdc42	Co-IP (Li et al., 2012)	No	No
“	Cla4	Co-IP (Li et al., 2012)	No	No
Rpp2A	Rpp2A	Y2H (Abramczyk et al., 2004)	No	Yes
“	Rpp2B	Y2H (Abramczyk et al., 2004)	No	Yes
Rpp2B	Rpp2A	Y2H (Abramczyk et al., 2004)	Yes	Yes
“	Rpp2B	Y2H (Abramczyk et al., 2004)	No	Yes
Cgt1	Cgt1	Y2H (Yamada-Okabe et al., 1998)	No	No
Ras2	Cyr1 (RA-domain)	Y2H (Fang and Wang, 2006)	No	Yes
Ras1	Cyr1	Aff C-MS (Wang et al., 2010b)	No	No
Ras1	Cyr1	Aff C-MS (Zou et al., 2010)	No	No
Hsp90§	Crk1	Y2H (Ni et al., 2004)	No	No
Hsp90§	Sti1	Y2H (Ni et al., 2004)	Yes	Yes
Hsp90§	Cdc37	Y2H (Ni et al., 2004)	Yes	Yes
Vrp1	Hof1	Y2H (Borth et al., 2010)	Yes	Yes
Opi1	Ino2	GST pulldown (Hoppen et al., 2007)	Yes	Yes
Opi1	Ino4	GST pulldown (Hoppen et al., 2007)	Yes	Yes
Opi1	Sin3	Y2H/GST pulldown (Heyken et al., 2003)	Yes	Yes
Far1^	Tpk1^	Co-IP (Yi et al., 2011)	No	No
Cst5^	Tpk1^	Co-IP (Yi et al., 2011)	No	No
Bas1	Gfr10	C2H (Wangsanut et al., 2018)	Yes	Yes
Gfr10	Bas1	C2H (Wangsanut et al., 2018)	Yes	Yes
Swi6	Nrm1	Co-IP (Ofir et al., 2012)	/	/

^∧^Are two interactions not expected to occur, they were used as negative controls. ^*^ Shows an interaction that was shown by Yi et al. (2011) with a Co-IP. ^∗∗^ Interaction demonstrated by Legrand et al. (2018) and Stynen et al. (2010). ° Interaction demonstrated by Stynen et al. (2010). §  It is not certain whether Hsp90 was used as a bait or prey construct (Ni et al., 2004). Cyt. Y2H = cytoplasmic Y2H. For Cek2 we used the “best hit” protein ScFus3 to compare the data from *C. albicans* with *S. cerevisiae*.

Apart from helping elucidate differences between two organisms, the “non-interacting” protein pairs might also indicate which technique could be used to study certain proteins or interactions.

### Case Study Regarding PPIs Shown in *C. albicans*

**FIGURE 2:**
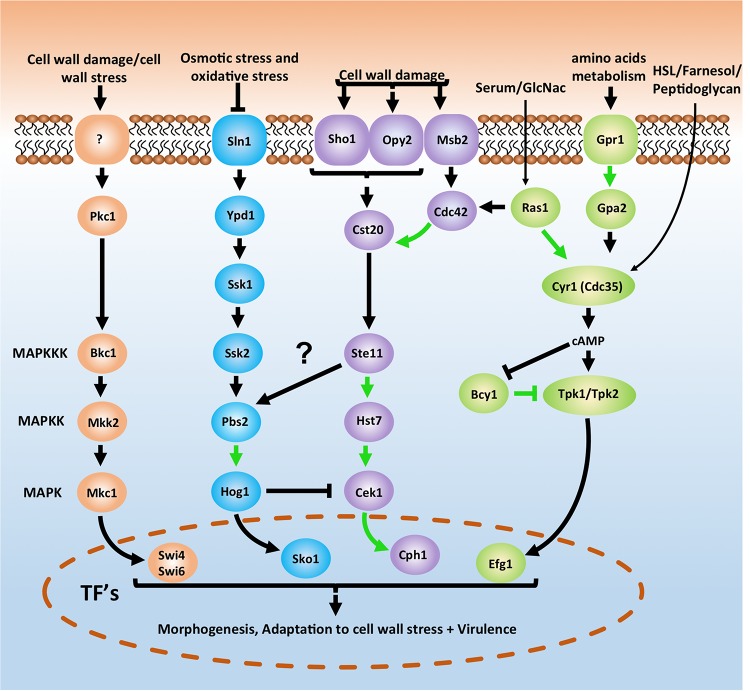
Basic schematic representation of the cAMP-PKA pathway (green) and three MAPK pathways in *Candida albicans* which are important for morphogenesis, adaptation to stress and survival. The cell integrity pathway (also known as PKC pathway) is depicted in orange, the HOG pathway is shown in blue and the CEK1 mediated pathway (also known as SVG pathway) is represented in purple. Pathways depicted here are an oversimplified version. A direct interaction between Msb2 and Cst20 (van Wijlick et al., 2016) is for example not depicted here. For a more in depth overview see Refs. (Biswas et al., 2007; de Dios et al., 2010; Sudbery, 2011; Huang, 2012; Noble et al., 2017; Burch et al., 2018). Notice also the arrow between Ste11 and Pbs2. In yeast the Sho1 branch plays a role in osmotic stress signaling to Hog1 (Hohmann, 2002). However, in *C. albicans* this does not seem to be the case as Ssk2 is the only MAPKKK signaling to Hog1 (Cheetham et al., 2007; Román et al., 2009). Arrows in green depict physical interactions between two proteins that have already been demonstrated in *C. albicans*. Arrows in black are Protein-protein interactions not yet demonstrated in *C. albicans*. See text for more details.

The cell wall is a dynamic structure that offers a first line of defense against external influences. As *C. albicans* can be present in any host niche, it must have a huge range of possible adaptations to external stresses (Ene et al., 2015; Román et al., 2016). Several pathways have been documented that play a role in these adaptation processes. Four of the most studied and important pathways are depicted in a simplified version in Figure 2. The cAMP-PKA pathway acts through a Ras1-independent or -dependent mechanism as a response to several external influences such as the quorum-sensing molecules homoserine lactone (HSL) and farnesol, amino acids, CO_2_, serum, *N*-acetylglucosamine (GlcNac) or glucose. The HOG pathway is important to react upon osmotic and oxidative stress while the cell wall integrity (PKC) and Cek1-mediated (or SVG) pathways play a role in the response toward cell wall damage and stress. All four pathways play a crucial role in morphogenesis and the survival of *C. albicans* under stress conditions (Biswas et al., 2007; de Dios et al., 2010; Noble et al., 2017). While there is considerable knowledge regarding how the pathways function in *C. albicans*, there is still a lot to demonstrate in terms of PPIs. Figure 2, for example, shows that only 8 out of the 26 depicted potential PPIs have been demonstrated in *C. albicans* (Chatr-Aryamontri et al., 2017). A ninth PPI that could be shown in Figure 2 is the direct interaction between Msb2 and Cst20 (van Wijlick et al., 2016). In *S. cerevisiae* all interactions for the depicted pathways in Figure 2 have already been demonstrated (Cherry et al., 2012).

## Conclusion

Of the plethora of available techniques to study PPIs, only a select few have been used intensively for large-scale high-throughput screenings. Two of the most important techniques are the TAP-MS and Y2H system (De Las Rivas and Fontanillo, 2010; Podobnik et al., 2016). Both techniques have been used to examine a large number of PPIs for several organisms, leading to a profound knowledge on the studied organisms and even the start of the construction of the interactome of the studied organisms (Ito et al., 2001; von Mering et al., 2002). PPI studies in *C. albicans* are, however, lagging behind. *C. albicans* is comparable with *S. cerevisiae* in terms of ORFs, respectively, 6,198 vs. 6,572. Both organisms serve as model organisms and are fully sequenced. However, a staggering 70% of the ORFs are still not characterized in *C. albicans* versus 12% in *S. cerevisiae* (Cherry et al., 2012; Skrzypek et al., 2017). Looking at PPIs the difference is even more extreme (based on release 3.5.174, July 2019); 1,080 interactions for *C. albicans* are mentioned in the BioGRID vs. 171,959 for *S. cerevisiae* (Chatr-Aryamontri et al., 2017; Oughtred et al., 2018). Supplementary Tables S1, S2 give an overview of interactions found in *C. albicans* that also have been detected in *S. cerevisiae* using orthologs. Supplementary Table S2 also shows that the majority of PPIs shown in *C. albicans* have been demonstrated with only one technique while in *S. cerevisiae* the majority of PPI have been demonstrated using multiple techniques. Interactions should, ideally, always be validated with two or more techniques (Auerbach et al., 2002; Gavin et al., 2002; von Mering et al., 2002). Despite being one of the first fully sequenced fungal pathogens (Jones et al., 2004), the difficulties encountered when working with *C. albicans* have slowed down the progress (Noble and Johnson, 2007). This is mainly due to the non-canonical codon usage in *C. albicans* and the long-held misconception that the gene function of *C. albicans* is very similar to that of *S. cerevisiae*. The latter has, however, been shown to be more complicated (Boysen et al., 2009). Until now, only 249 out of a total of 1,208 non-redundant PPIs in *C. albicans* have also been demonstrated in *S. cerevisiae* (see also Supplementary Tables S1, S2). A critical note here is that our tables also contain PPIs demonstrated in other *C. albicans* strains than the wild type-strain SC5314 (or its derivative strains) and PPIs deposited at the rcsb protein structure database, but not yet published.

Knowing that a similar organism, *S. cerevisiae*, has an estimated total of 30,000 to 40,000 interactions (Grigoriev, 2003; Sambourg and Thierry-Mieg, 2010) or even more (Hart et al., 2006), there is still a lot to discover for *C. albicans*. Besides giving fundamental knowledge, PPIs can also be used as very specific drug targets (Khan et al., 2011). Previously thought undruggable, PPIs have become increasingly interesting targets for drug development (Modell et al., 2016). Given the limited availability of antifungals, rising resistance, lack of antifungal vaccines, difficulties in antifungal drug development, and the increase of fungal infections worldwide (Brown et al., 2012; Fairlamb et al., 2016, Fairlamb et al., 2017; Patin et al., 2018) PPIs might become crucial in the future development of novel, specific antifungals.

In the “omics” era, the enormous amount of information generated by a wide range of large-scale, high-throughput assays creates severe problems for data storage and sorting, which emphasizes the importance of data collection and curation (Costanzo et al., 2006; Reguly et al., 2006). Open databases such as the *Candida* genome database (CGD) (Skrzypek et al., 2017) and the BioGRID (Oughtred et al., 2016, 2018; Chatr-Aryamontri et al., 2017) are crucial tools for *Candida* researchers. Integration of the BioGRID PPI dataset into the CGD would be a substantial improvement of the CGD. Currently the CGD only mentions the BioGRID as an external link on the summary page of the genes.

While using PPI data from *S. cerevisiae* to aid in constructing *C. albicans* PPI networks, caution is advised considering the low overlap between the PPI data (Supplementary Tables S1, S2). Das et al. (2019), for example, constructed a PPI network with a focus on proteins important for hyphae formation in *C. albicans* using data from *S. cerevisiae*, a species generally regarded as only forming pseudohyphae, as a control for the validation of interactions (Arkowitz and Bassilana, 2011; Das et al., 2019). Comprehensive, high-quality databases of *C. albcians* genome sequences and PPIs will make it possible to resolve the *C. albicans* interactome based on *C. albicans* data rather than inferences from data obtained in *S. cerevisiae* (Fraser et al., 2003; Tsai et al., 2014; Wang et al., 2014; Márkus et al., 2017; Das et al., 2019). PPI databases, such as the BioGRID, play a crucial role in elucidating the protein interaction networks but also rely on external help to grow and keep up with the most recent research (the BioGRID relies on researchers to send in their data as it does not actively track PPI data from *C. albicans*). The latter was noticeable by the absence of a huge number of interactions in the BioGRID at the start of this review (compare releases 3.4.156 and 3.5.174), yet it is of the utmost importance for researchers to send in their data as the whole research community depends on such databases to perform their experiments with a high-quality dataset (Costanzo et al., 2006; Cusick et al., 2009; Oughtred et al., 2016).

With an exhaustive literature search, we tried to include most of the PPIs known in *C. albicans* in the BioGRID dataset but are aware that some might still be missing. We therefore hope that future studies on PPIs in *C. albicans* will be sent to databases such as the BioGRID (an excel template for submitting PPI data can be found here^5^) so that a more complete overview regarding PPIs can be achieved for *C. albicans*.

## Author Contributions

FS initiated this review, compiled all the data, provided all the input to BioGRID, and wrote the draft version of the manuscript. FS and PV wrote the final version of the manuscript.

## Conflict of Interest Statement

The authors declare that the research was conducted in the absence of any commercial or financial relationships that could be construed as a potential conflict of interest.

## Footnotes

1 https://www.rcsb.org

2 http://www.candidagenome.org

3 https://www.yeastgenome.org

4 https://thebiogrid.org

5 https://wiki.thebiogrid.org/lib/exe/fetch.php/biogrid_data_submission_form.xls
